# Long-Lasting Antibody and CD8^+^ Memory T Cell Responses Induced by N-Tc52/TSKb20 Vaccination upon *Trypanosoma cruzi* Antigen Re-Encounter

**DOI:** 10.3390/vaccines14060526

**Published:** 2026-06-13

**Authors:** María Elisa Vázquez, Brenda A. Zabala, Maria Constanza Barrientos, Daniela E. Barraza, María A. Occhionero, Federico Ramos, Alejandro Uncos, Leonardo Acuña, Cecilia Pérez Brandán

**Affiliations:** Unidad de Biotecnología y Protozoarios (UBIPRO), Instituto de Patología Experimental “Dr. Miguel Ángel Basombrío” (IPE), Consejo Nacional de Investigaciones Científicas y Técnicas (CONICET), Universidad Nacional de Salta (UNSa), Salta A4400, Argentina; elisa.vazquez@conicet.gov.ar (M.E.V.); b.zabala@conicet.gov.ar (B.A.Z.); mcontibarrientos@conicet.gov.ar (M.C.B.); barrazadaniela@conicet.gov.ar (D.E.B.); mariaocchionero@conicet.gov.ar (M.A.O.); federamosqui@gmail.com (F.R.); auncos54@gmail.com (A.U.)

**Keywords:** *Trypanosoma cruzi*, long-lasting immunity, chimeric protein, N-Tc52/TSKb20, immunodominant domains

## Abstract

**Background**: Chagas disease, caused by *Trypanosoma cruzi*, remains a major public health problem in Latin America and an emerging concern worldwide. Current chemotherapies show limited efficacy during chronic infection, and no licensed vaccine is currently available. We previously developed the chimeric antigen N-Tc52/TSKb20 as a vaccine candidate against *T. cruzi* infection. In a murine model, this vaccine induced robust antigen-specific immune response associated with protection shortly after vaccination. **Objectives**: Here, we investigated the long-term persistence and effector functions of the immune responses elicited by this vaccine candidate. **Methods**: Both female and male C57BL/6 mice were immunized with three doses of N-Tc52/TSKb20 formulated with QuilA adjuvant. Serum samples collected 170 days post-immunization were analyzed for antigen-specific antibodies by ELISA and for trypanolytic activity against cell-derived trypomastigotes using an in vitro functional assay. Cellular immune responses were evaluated by measuring cytokine production, T cell activation, and memory T cell responses following in vitro re-stimulation with the vaccine antigen or *T. cruzi* antigens. **Results**: N-Tc52/TSKb20 vaccination induced a sustained antigen-specific humoral response, characterized by long-lasting IgG2c antibodies and functional activity persisting for up to 170 days post-immunization. In parallel, vaccination promoted long-term activation of antigen-specific CD8^+^ T cells and production of TNF-α and IFN-γ upon antigen re-encounter. A sex-dependent tendency was observed for IL-10, with increased production in vaccinated female mice. Moreover, vaccinated animals exhibited increased frequencies of central and effector memory CD4^+^ and CD8^+^ T cells in response to *T. cruzi* antigens, with a predominant contribution of CD8^+^ T cells, indicating the establishment of parasite-specific T cell memory. **Conclusions**: Together, these findings demonstrate that vaccination with N-Tc52/TSKb20 induces a long-lasting Th1-biased immune response characterized by trypanolytic antibodies, functional and durable T cell responses, and parasite-specific memory T cells. This immunological profile supports the potential of N-Tc52/TSKb20 as a promising vaccine candidate for Chagas disease and highlights its capacity to elicit immune mechanisms that have been associated with protection against *T. cruzi* infection.

## 1. Introduction

Vaccination remains one of the most powerful tools in public health, preventing millions of deaths each year by priming the immune system to recognize and eliminate pathogens before disease onset [1]. However, the true success of vaccination depends not only on the magnitude of the initial immune response but also on its durability. Long-lasting immune protection, mediated by long-lived plasma cells, memory B cells, and memory T lymphocytes, enables the immune system to mount rapid and effective effector responses upon pathogen encounter without the need for frequent booster doses [2,3]. These durable responses are critical for vaccine effectiveness, as they allow early control of infection, thereby preventing severe disease, reducing morbidity and mortality, and limiting pathogen transmission [4,5].

Licensed human vaccines confer varying degrees of long-term protection. While some provide lifelong immunity, others require booster doses at defined intervals, and some must be administered annually [5]. This variability in the durability of vaccine-induced protection underscores the importance of evaluating the persistence of immune responses as a formulation-dependent feature. Despite its relevance, the mechanisms underlying long-term immunity following vaccination remain incompletely understood [3]. In this context, many infectious diseases caused by microorganisms still lack a licensed vaccine. Chagas disease (CD), caused by the protozoan parasite *Trypanosoma cruzi* (*T. cruzi*), is one such example. CD remains one of the most neglected tropical diseases, silently affecting more than six million people worldwide. Originally confined to Latin America, it is increasingly emerging beyond endemic regions as a consequence of global migration, with transmission now occurring predominantly through non-vectorial routes such as vertical infection, blood transfusion, and organ transplantation [6,7,8]. *T. cruzi* infection represents a complex immunological challenge: the parasite exhibits remarkable antigenic diversity, persistence strategies and immune evasion mechanisms that subvert both innate and adaptive immune responses, resulting in a delicate balance between parasite control and tissue damage [9,10]. Additionally, the limited understanding of the pathophysiology of CD and the complex host–parasite interactions continue to hinder the development of an effective vaccine against *T. cruzi* [11]. Various vaccine strategies have been explored, ranging from early approaches based on inactivated or attenuated parasites to the evaluation of purified parasite antigens [12,13,14,15,16,17,18]. More recently, recombinant proteins and subunit vaccines have emerge as promising alternatives [19,20,21,22,23,24,25]. Current vaccine design increasingly focuses on incorporating multiple epitopes that target distinct immune pathways, aiming to enhance both the breadth and magnitude of the protective immune response [26,27,28,29,30]. The outcomes and limitations of these different approaches have been extensively reviewed elsewhere [11,31,32,33,34].

In the development of vaccines against *T. cruzi*, protective immune responses must persist long after immunization to ensure rapid control of parasite infection [35]. To date, no validated correlates of protection reliably predict vaccine efficacy against CD [36,37,38]. However, studies in experimental models and infected individuals have identified key immune mechanisms involved in parasite control, particularly cellular responses associated with protection against intracellular pathogens [39]. Although these responses are widely used as reference parameters to evaluate candidate vaccines, they do not constitute definitive predictors of efficacy. In this context, the induction and maintenance of durable immune memory emerge as fundamental features of effective vaccination [40,41]. Therefore, assessing the persistence of immune responses associated with protection represents a critical component in the development and evaluation of vaccine strategies against *T. cruzi*.

Recent work from our group has provided compelling evidence that the rational combination of immunodominant parasite domains within a recombinant chimeric protein represents a powerful strategy to enhance immunogenicity and protective efficacy [42]. Among the selected components of our recombinant chimera is the amino-terminal domain of the *T. cruzi* Tc52 protein, which has been shown to induce functional antibodies associated with partial protection against infection [20]. The second component is TSKb20, an immunodominant CD8^+^ T cell epitope derived from the Trans-sialidase family, whose antigen-specific cellular responses have also been associated with protection in experimental models [43]. Vaccination with the N-Tc52/TSKb20 chimera previously demonstrated the ability to elicit robust and coordinated humoral and cellular immune responses shortly after immunization. These responses were characterized by the induction of IgG2c antibodies and the activation of TSKb20-specific CD8^+^ T cells and were associated with effective control of *T. cruzi* infection following challenge, resulting in superior protection compared with either antigenic domain alone [42]. Collectively, these findings highlight the potential of N-Tc52/TSKb20 to drive functional and protective immunity against *T. cruzi*.

In the present study, we sought to define the durability and functional capacity of the immune responses elicited by the N-Tc52/TSKb20 antigen 170 days after the last immunization, focusing on immunological memory as a hallmark of effective vaccination. We performed a comprehensive evaluation of both humoral and cellular responses in a C57BL/6 murine model at approximately six months after the final immunization. Given the dual extracellular and intracellular nature of *T. cruzi*, this integrated approach enabled the characterization of immune mechanisms required for sustained parasite control. Altogether, these analyses provide critical insights into the longevity and functional competence of the immune response induced by the N-Tc52/TSKb20 chimera, further reinforcing its potential as a promising vaccine candidate against CD.

## 2. Materials and Methods

### 2.1. Animal Model

C57BL/6 female and male mice (8 weeks old) were used throughout these experiments. Animals were housed in groups of up to five per cage under controlled temperature (25 °C) and a 12 h light/12 h dark photoperiod, with ad libitum access to standard feed and water. At the end of the experiments, animals were euthanized by controlled carbon dioxide (CO_2_) exposure to minimize suffering. Animal breeding and experimental procedures were conducted at the Animal Facility of the Instituto de Patología Experimental, Universidad Nacional de Salta, Argentina. All procedures were reviewed and approved by the Comité Institucional para el Cuidado y Uso de Animales de Laboratorio (CICUAL) of the Facultad de Ciencias de la Salud, Universidad Nacional de Salta (Salta, Argentina). The study was approved under Resolution CD No. 093-22 (Expedient No. 12.092/21), issued on 4 April 2022.

### 2.2. Immunization Protocol

Mice (*n* = 7; 3 females and 4 males) were immunized subcutaneously with three doses of 20 µg of N-Tc52/TSKb20 combined with 15 μg of the QuilA adjuvant (InvivoGen, Toulouse, France), administrated in 50 µL injections every 3 weeks. A non-vaccinated control group received 15 μg of QuilA alone (*n* = 6; 3 females and 3 males) and was included throughout the study. On day 170 post-vaccination, animals were euthanized following ethical standards to assess humoral and cellular responses.

### 2.3. Detection of N-Tc52/TSKb20-Specific Antibodies

For antibody determination assays, serum samples were collected from vaccinated and non-vaccinated mice at 170 days after the final dose. Sera were obtained by blood centrifugation at 3500 rpm, which were subsequently stored at −20 °C until IgG1 and IgG2c evaluation; 96-well plates were coated with 0.25 μg/well of the chimeric protein in 100 µL of Coating Buffer and incubated overnight at 4 °C. IgG1 and IgG2c subclasses were determined by ELISA. Plates were blocked with 5% skim milk in PBS for 1 h at 37 °C and then incubated with sera diluted 1:20 (100 μL/well) for 1 h at 37 °C. Biotinylated anti-IgG1 or anti-IgG2c antibodies (BD Biosciences, San José, CA, USA) were added at dilutions of 1:4000 and 1:2000, respectively, and incubated for 1 h at 37 °C. Horseradish peroxidase-conjugated streptavidin (1:7500) was subsequently added and incubated for 1 h at 37 °C. Signal development was performed with 1 mg/mL of o-Phenylenediamine dihydrochloride OPD (Merck, Darmstadt, Germany) with a dilution 1:10 of 30% p/v H_2_O_2_ 30% for 20 min and stopped with 2 N H_2_SO_4_. Optical density was measured at 492 nm using a TECAN Infinite F50 plate reader (Thermo Fisher Scientific, Waltham, MA, USA).

### 2.4. Cell Culture-Derived Trypomastigote and Trypanolytic Activity

Cell culture-derived trypomastigotes of the Tulahuen strain (DTU VI) were obtained as follows. Epimastigotes were maintained in vitro through routine passages and cultured at 28 °C in Liver Infusion Tryptose (LIT) medium supplemented with 20 µg/mL hemin (Sigma, St. Louis, MO, USA), 100 IU/mL penicillin, 100 µg/mL streptomycin, and 10% heat-inactivated fetal bovine serum at 56 °C for 1 h (FBS; Natocor, Córdoba, Argentina). To induce differentiation into metacyclic trypomastigotes, cultures were subjected to nutrient starvation for 3 weeks without medium supplementation, followed by overnight incubation at 37 °C. Metacyclic trypomastigotes were collected by centrifugation at 1500 rpm for 10 min, washed twice with PBS, and used to infect L929 cells at 60% confluence. Infected cells were cultured in DMEM at 37 °C with 5% CO_2_ for 15 days, during which cell culture-derived trypomastigotes were released into the media. Trypomastigotes were then collected by centrifugation, washed, and used to assess antibody-mediated parasite lysis.

To evaluate trypanolytic activity, sera from vaccinated and non-vaccinated mice were heat-inactivated at 56 °C 30 min to abolish endogenous complement activity. Subsequently, 40 µL of serum was mixed with 2 × 10^5^ cell culture-derived trypomastigote and 40 µL of human serum diluted 1:2 as an exogenous complement source, then incubated 3 h at 37 °C. Under these conditions, the assay specifically assessed the capacity of vaccine-induced antibodies to promote lysis of *T. cruzi* trypomastigotes in the presence of active human complement. The percentage of trypomastigote lysis was determined by counting live parasites in 1:10 diluted samples using a Neubauer chamber (Boeco, Hamburg, Germany) under light microscopy at 1 and 3 h post-incubation. All measurements were performed in duplicate.

### 2.5. Splenocyte Culture

Spleens were harvested from euthanized mice on day 170 post-vaccination and placed on 15 mL tubes containing Roswell Park Memorial Institute (RPMI 1640) medium supplemented with L-glutamine (Biological Industries, Beit Haemek, Israel). Spleens were then gently macerated through a sterile mesh on ice, and the resulting cell suspension was collected by centrifugation at 160× *g* at 4 °C for 10 min. Erythrocytes were removed by resuspending the pellet in lysis solution (0.17 M Tris, 0.16 M NH_4_Cl, pH 7.2). The remaining splenocytes were washed and resuspended in RPMI supplemented with 10% FBS that was heat-inactivated. Cell number and viability were assessed by Trypan blue exclusion using a Neubauer chamber.

### 2.6. Tulahuen HP Preparation

A total soluble protein homogenate from *T. cruzi* Tulahuen strain (Tulahuen HP) was prepared from a culture of 1 × 10^9^ epimastigotes in the exponential growth phase. Parasites were maintained in LIT medium supplemented with 20 µg/mL hemin, 100 IU/mL penicillin, 100 µg/mL streptomycin, and 10% heat-inactivated FBS at 56 °C for 1 h. Parasites were then centrifuged at 1000× *g*, washed, and resuspended in a lysis buffer (100 mM Tris-HCl, 1 mM EDTA, pH 8). The suspension was incubated at 4 °C for 10 min. Parasite lysis was completed by three freeze–thaw cycles followed by sonication (three pulses of 1 min at 40 W, 80% amplitude) and confirmed by microscopic observation. The lysate was then centrifuged at 20,000× *g* at 4 °C for 20 min. The supernatant containing soluble parasite proteins was collected and sterilized by filtration through a 0.22 µm membrane. Protein homogenates were stored at −70 °C until use.

### 2.7. Activation-Induced Marker Assay (AIM)

The frequency and activation of specific CD8^+^ and CD4^+^ T cells were evaluated using an activation-induced marker (AIM) assay in splenocytes obtained at 170 days post-vaccination [42,44]. Splenocytes (1 × 10^6^ cells/well) were stimulated for 15 h with 0.2 μM of the chimeric protein, 0.4 μM of the TSKb20 synthetic peptide (GenScript, Piscataway, NJ, USA) or 15 μg/mL of Tulahuen HP. Unstimulated cells (culture medium) served as negative controls, and cells stimulated with Concanavalin A (CoA, 5 μg/mL, Merck) were used as positive controls. After incubation, cells were washed with PBS and stained with anti- IA/IE-APCCy7 (clone m5/114.15.2), anti-B220-APCCy7 (clone RA3-6B2) y Zombie Fixable Viability near-IR dye (exclusion/DUMP panel), followed by anti-CD8-PerCP (clone 53-6.7), anti-CD4-FITC (clone GK15), anti-CD25-APC (clone PC61) and anti-CD69-PECy7 (clone H1.2F3) (Biolegend, San Diego, CA, USA) for 30 min at 4 °C in the dark. Cells were then fixed with 4% formaldehyde for 16 h at 4 °C, washed with PBS and acquired on a BD FACS Canto II cytometer. Data were analyzed using FlowJo vX.0.7.

### 2.8. Cytokine Quantification

Supernatants from splenocyte cultures (1 × 10^6^ cells/mL), collected after 15 h of stimulation with 0.2 μM N-Tc52/TSKb20, 0.4 μM TSKb20 peptide or 15 μg/mL Tulahuen HP, were used to measure TNF-α and IL-10 levels using the Mouse cytokine ELISA MAX™ Standard Set (BioLegend) following the manufacturer’s instructions. Briefly, plates were coated overnight with capture antibody (1:200), blocked with 10% FBS, and incubated for 2 h with the samples. Detection antibody (1:200) was added and incubated for 1 h at room temperature. After washes, horseradish peroxidase (1:1000) was added and incubated for 30 min. Signal development was performed for 30 min with 1 mg/mL OPD and 1:10 diluted 30% H_2_O_2_, and the reaction was stopped with 2 N H_2_SO_4_. Absorbance was read at 492 nm using a TECAN plate reader. The limits of detection of the assays were 7.8 pg/mL and 31.3 pg/mL for TNF-α and IL-10, respectively.

### 2.9. ELISPOT Assay for IFN-γ Detection

An ELISPOT assay (BD Biosciences) was performed to quantify IFN-γ-producing splenocytes from mice euthanized 170 days post-vaccination, following the manufacturer’s instructions. Briefly, ELISPOT plates were coated overnight with anti-IFN-γ capture antibody and blocked for 2 h at room temperature. Splenocytes (1 × 10^6^ cells/well) were seeded and stimulated with 0.2 μM N-Tc52/TSKb20 protein, 0.4 μM TSKb20 peptide, or 15 μg/mL Tulahuen HP for 24 h at 37 °C with 5% CO_2_. After incubation, plates were washed and incubated with a biotinylated anti-IFN-γ detection antibody, followed by streptavidin–horseradish peroxidase. Spots were developed using 3,3′-diaminobenzidine tetrahydrochloride (DAB; Merck) and 1:10 diluted 30% H_2_O_2_. IFN-γ-secreting cells were quantified using an ImmunoSpot S5.0.3 reader at the Instituto Fatala Chabén, Buenos Aires, Argentina. Unstimulated cells (culture medium) served as negative controls, and cells stimulated with CoA (5 μg/mL; Merck) were used as positive controls.

### 2.10. Identification of Specific Immunological Memory Subpopulations

To characterize antigen-specific CD4^+^ and CD8^+^ memory T-cell populations, spleens were collected 170 days post-vaccination and processed for flow cytometry analysis. Splenocytes (1 × 10^6^ cells/well) were stimulated for 15 h with 0.2 μM of the chimeric protein, 0.4 μM of the TSKb20 peptide, or 15 μg/mL of Tulahuen HP. Unstimulated cells (culture medium) served as negative controls, and cells stimulated with CoA (5 μg/mL; Merck) were used as positive controls. Following incubation, cells were washed with PBS and stained with anti-IA/IE-APCCy7 (clone m5/114.15.2), anti-B220-APCCy7 (clone RA3-6B2) and Zombie Fixable Viability near-IR dye (exclusion/DUMP panel), followed by anti-CD8-PerCP (clone 53-6.7), anti-CD4-FITC (clone GK15), anti-CD62L-APC (clone MEL-14) and anti-CD44-PECy7 (clone IM7) (Biolegend) for 30 min at 4 °C in the dark. Cells were then fixed with 4% formaldehyde for 16 h at 4 °C, washed with PBS, and acquired on a BD FACS Canto II cytometer. Data were analyzed using FlowJo vX.0.7.

### 2.11. Statistical Analysis

Data distribution was assessed using the Shapiro–Wilk normality test prior to statistical analysis. Data were then analyzed using two-way analysis of variance (ANOVA) followed by Sidak’s multiple-comparisons test. Results are presented as mean ± standard error of the mean (SEM). All statistical analyses were performed using GraphPad Prism version 8.0.2 (GraphPad Software, San Diego, CA, USA). Differences were considered statistically significant when *p* < 0.05.

## 3. Results

### 3.1. N-Tc52/TSKb20 Vaccination Induces Durable IgG2c-Biased Antibody Responses with Trypanolytic Activity

**Figure 1:**
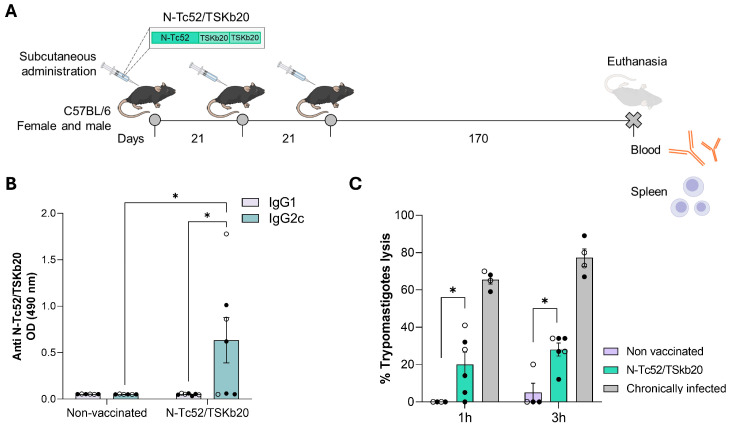
Long-term presence of N-Tc52/TSKb20-specific IgG2c antibodies with trypanolytic activity in sera from vaccinated mice. (**A**) Schematic representation of the immunization protocol in female and male C57BL/6 mice. Animals received three subcutaneous doses of 20 µg of N-Tc52/TSKb20 formulated with 15 µg of QuilA at 21-day intervals. Non-vaccinated mice were used as controls. To evaluate long-term immune responses, mice were euthanized 170 days after the last immunization, and blood and spleen samples were collected for the analysis of humoral and cellular immune responses, respectively. (**B**) Serum levels of anti-N-Tc52/TSKb20 IgG1 and IgG2c antibodies were determined by ELISA. (**C**) Trypanolytic activity of sera against *T. cruzi* trypomastigotes after 1 h and 3 h of in vitro incubation. Sera from chronically infected mice were used as positive controls. Each symbol represents an individual mouse; open circles correspond to females and filled circles to males. Data are presented as individual values with mean ± SEM. Statistical analysis was performed using two-way ANOVA followed by Sidak’s multiple comparisons test. Asterisks indicate statistically significant differences (* *p* < 0.05).

To evaluate the humoral immune response, female and male C57BL/6 mice were immunized following the vaccination protocol described in Figure 1A. Antigen-specific IgG1 and IgG2c levels were measured 170 days after the final immunization. Two-way ANOVA analysis revealed no significant interaction between sex and vaccination, indicating that the effect of vaccination on the humoral response was independent of sex. Accordingly, data from both sexes were analyzed together. Mice vaccinated with N-Tc52/TSKb20 exhibited significant levels of antigen-specific IgG2c antibodies against the chimeric protein, whereas IgG1 levels were undetectable (*p* < 0.05) (Figure 1B). The level of this isotype was significantly higher in vaccinated animals compared with non-vaccinated controls (*p* < 0.05) (Figure 1B). This profile was indicative of a sustained Th1-oriented humoral response. Notably, persistent IgG2c antibodies were detected in four vaccinated mice (two females and two males), highlighting inter-individual differences in the long-term humoral response.

We next assessed the functional capacity of immune sera to mediate in vitro trypomastigote lysis. As shown in Figure 1C, after 1 h of incubation, sera from vaccinated mice displayed significantly higher lytic activity than non-immune sera, with approximately 20% parasite lysis observed in nearly half of the samples (*p* < 0.05). After 3 h of incubation, trypanolytic activity further increased in most samples, with mean lysis levels approaching 30%, significantly exceeding those detected in non-immune sera (*p* < 0.05). No sex-dependent differences in trypanolytic activity were detected at either time point.

### 3.2. Immunization with the Chimeric Protein Promotes Long-Term Activation of TSKb20-Specific CD4^+^ and CD8^+^ T Cells

**Figure 2:**
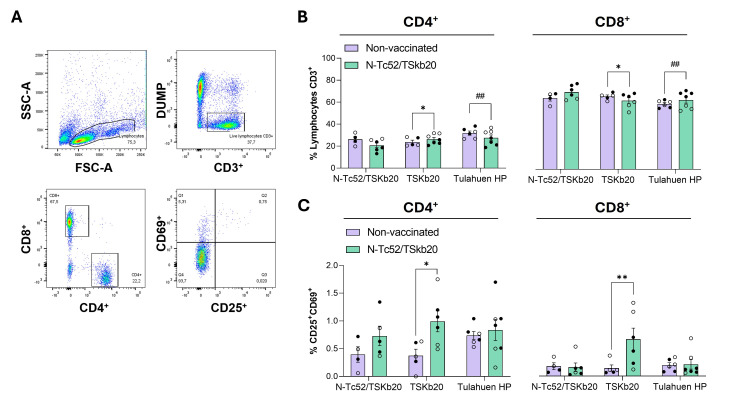
Persistence of TSKb20-specific CD4^+^ and CD8^+^ T cell responses in splenocytes from N-Tc52/TSKb20 vaccinated mice upon antigen re-stimulation. (**A**) Gating strategy used for activation-induced marker (AIM) analysis by flow cytometry. Splenocytes were collected 170 days after the last immunization and analyzed after 15 h of ex vivo stimulation with N-Tc52/TSKb20 protein, TSKb20 peptide, or *T. cruzi* protein homogenate (Tulahuen HP), to identify CD3^+^CD4^+^ and CD3^+^CD8^+^ T cell populations co-expressing the activation markers CD25^+^ and CD69^+^. (**B**) Percentages of CD3^+^CD4^+^ (left panel) and CD3^+^CD8^+^ (right panel) T cells. (**C**) Percentages of CD4^+^ (left panel) and CD8^+^ (right panel) T cells co-expressing the activation markers CD25 and CD69. Each symbol represents an individual mouse; open circles correspond to females and filled circles to males. Data are presented as individual values with mean ± SEM. Statistical analysis was performed using two-way ANOVA followed by Sidak’s multiple comparisons test. Symbols * and # indicate statistically significant differences (* *p* < 0.05; ** *p* < 0.01). For CD4^+^ and CD8^+^ T cell percentage analysis (**B**), statistical differences relative to the non-vaccinated group were performed separately for females (* *p* < 0.05) and males (## *p* < 0.01).

To evaluate the long-term cellular immune response, splenocytes were isolated 170 days after the last immunization and re-stimulated in vitro for 15 h with vaccine-related antigens, including the N-Tc52/TSKb20 protein and the TSKb20 peptide, as well as parasite-derived soluble proteins from *T. cruzi* (Tulahuen HP). The frequencies of CD4^+^ and CD8^+^ T cells and their activated subpopulations (CD25^+^CD69^+^) were determined by flow cytometry following the gating strategy shown in Figure 2A.

We first assessed whether changes in CD4^+^ and CD8^+^ T cell frequencies upon antigen recall were influenced by sex. Two-way ANOVA revealed a significant interaction between sex and vaccination when cells were stimulated with TSKb20 (*p* < 0.05) and Tulahuen HP (*p* < 0.01) for both CD4^+^ and CD8^+^ T cell populations. Therefore, subsequent analyses of T cell frequencies were performed separately for females and males. In male mice (filled circles), Tulahuen HP stimulation induced a shift in T cell subset distribution, characterized by a decreased frequency of CD4^+^ T cells (Figure 2B, left panel) and a concomitant increase in CD8^+^ T cells (Figure 2B, right panel) in vaccinated animals relative to non-vaccinated controls (*p* < 0.001). No significant differences were observed with the other stimuli. In contrast, the most evident differences in female mice (open circles) were observed following stimulation with the TSKb20 peptide. Vaccinated females exhibited an increased frequency of CD4^+^ T cell accompanied by a reduction in CD8^+^ T cells compared with non-vaccinated controls (*p* < 0.05) (Figure 2B), revealing a trend opposite to that observed in males following Tulahuen HP stimulation. No differences were detected in females with the remaining stimuli.

Antigen-specific T cell activation was subsequently assessed by measuring the co-expression of CD25 and CD69. Unlike the population frequency data, no significant interaction between sex and vaccination was detected in activated T cell subsets, indicating that the vaccine-induced activation response was not influenced by sex. Therefore, data from female and male mice were combined for further analysis. A consistent trend toward higher frequencies of activated CD4^+^CD25^+^CD69^+^ T cells was observed in vaccinated animals compared with non-vaccinated, irrespective of the antigen used for re-stimulation. This effect was particularly evident following stimulation with the TSKb20 peptide (*p* < 0.05) (Figure 2C, left panel). Importantly, within the CD8^+^CD25^+^CD69^+^ subpopulation, re-stimulation with the TSKb20 peptide elicited a significant increase in splenocytes from N-Tc52/TSKb20-vaccinated mice compared with the non-vaccinated group (*p* < 0.01) (Figure 2C, right panel), highlighting the persistence of antigen-specific CD8^+^ T cell responsiveness. Although no significant increase was detected following stimulation with the full N-Tc52/TSKb20 protein or Tulahuen HP, these findings collectively support the long-term maintenance of functional antigen-specific cellular immunity induced by vaccination.

### 3.3. Sustained Production of TNF-α and IFN-γ and Female-Biased IL-10 Response After TSKb20 Re-Exposure in Splenocytes from N-Tc52/TSKb20 Vaccinated Mice

**Figure 3:**
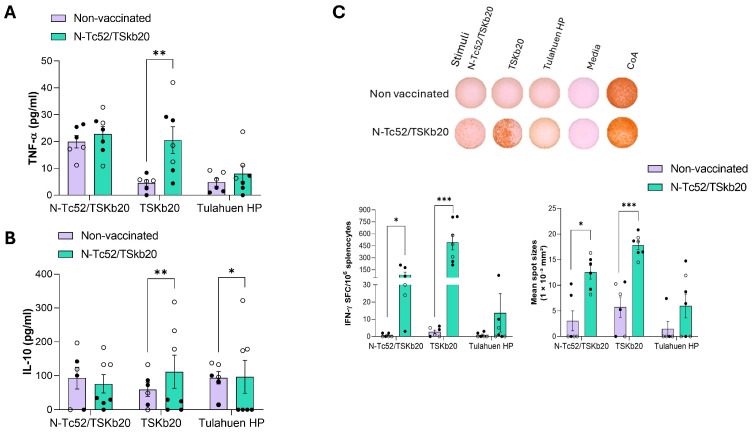
Sustained production of TNF-α and IFN-γ and female-biased IL-10 response in splenocytes from vaccinated mice after antigen re-encounter. Cytokine levels were measured in supernatants of splenocytes re-stimulated for 15 h with N-Tc52/TSKb20 protein, TSKb20 peptide, or *T. cruzi* protein homogenate (Tulahuen HP) by ELISA. (**A**) TNF-α levels. (**B**) IL-10 levels. (**C**) IFN-γ production was evaluated by ELISPOT after 24 h of stimulation with the indicated stimuli. The number of spot-forming cells (SFC) per 10^6^ splenocytes (left panel) and mean spot size (right panel) are shown. Representative images from each group are also included, along with positive (CoA) and negative (media) controls. Each symbol represents an individual mouse; open circles correspond to females and filled circles to males. Data are presented as individual values with mean ± SEM. Statistical analysis was performed using two-way ANOVA followed by Sidak’s multiple-comparisons test. Asterisks indicate statistically significant differences (* *p* < 0.05; ** *p* < 0.01; *** *p* < 0.001). For IL-10 analysis (**B**), statistical comparisons were performed separately by sex; significant differences were observed only in females and are indicated by asterisks.

To evaluate the durability of the effector response, cytokine production was measured in supernatants from stimulated splenocytes. TNF-α production was not influenced by sex, as no significant interaction between sex and vaccination was detected. Therefore, data from male and female mice were analyzed together. Vaccinated mice exhibited increased TNF-α production compared to non-vaccinated upon re-stimulation with the TSKb20 peptide (*p* < 0.01). In contrast, no differences in TNF-α levels were observed following stimulation with the full N-Tc52/TSKb20 protein or Tulahuen HP (Figure 3A).

In contrast, IL-10 production revealed a significant interaction between sex and vaccination status following stimulation with the chimeric protein (*p* < 0.05) as well as with TSKb20 and Tulahuen HP (*p* < 0.01). Accordingly, male and female mice were analyzed separately. A significant increase in IL-10 production was observed in supernatants from vaccinated female mice following stimulation with N-Tc52/TSKb20 (*p* < 0.01) and *T. cruzi* proteins (*p* < 0.05) compared with non-vaccinated females. This effect was not observed in males, as IL-10 levels remained low and comparable between vaccinated and non-vaccinated animals following antigen re-stimulation (Figure 3B).

In parallel, IFN-γ responses were evaluated by ELISPOT. Similar to TNF-α, IFN-γ production showed no significant interaction between sex and vaccination status. Therefore, data from male and female mice were pooled for subsequent analyses. Mice vaccinated with N-Tc52/TSKb20 exhibited a higher frequency of IFN-γ–producing splenocytes following re-stimulation with either the chimeric protein (*p* < 0.05) or the TSKb20 peptide (*p* < 0.001), compared with non-vaccinated controls (Figure 3C, left panel). In contrast, although no statistically significant differences were detected following stimulation with Tulahuen HP, vaccinated animals consistently showed a trend toward higher frequencies of IFN-γ-producing cells. Notably, splenocytes from vaccinated animals re-stimulated with the chimeric protein (*p* < 0.05) or the TSKb20 peptide (*p* < 0.001) exhibited significantly larger IFN-γ spots, consistent with greater cytokine secretion *per* responding cell. A similar trend toward increased spot size was observed following Tulahuen HP stimulation, although this did not reach statistical significance (Figure 3C, right panel). Representative ELISPOT images from each experimental group, including positive (CoA) and negative (medium) controls, are shown in Figure 3C.

### 3.4. Induction of Specific CD4^+^ and CD8^+^ T Cell Memory Subpopulations by N-Tc52/TSKb20 Vaccination

**Figure 4:**
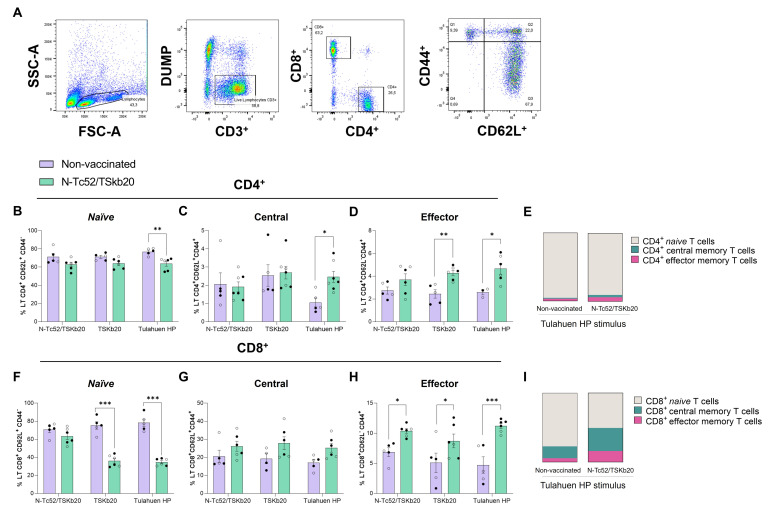
Vaccination with N-Tc52/TSKb20 induces central and effector memory T cell subsets upon *T. cruzi* antigen re-encounter. (**A**) Gating strategy used for flow cytometry analysis of CD4^+^ and CD8^+^ T memory T cell subsets based on CD44 and CD62L expression. Splenocytes were collected 170 days after the last immunization, cultured, and stimulated for 15 h with N-Tc52/TSKb20 protein, TSKb20 peptide, or *T. cruzi* protein homogenate (Tulahuen HP). (**B**) Frequencies of naïve (T_N_: CD62L^+^CD44^−^), (**C**) central memory (T_CM_: CD62L^+^CD44^+^), (**D**) and effector memory (T_EM_: CD62L^−^CD44^+^) subsets within CD3^+^CD4^+^ T cells. (**E**) Relative distribution of CD4^+^ T_N_, T_CM_, and T_EM_ subsets, expressed as proportions of their combined total after Tulahuen HP stimulation. (**F**) Frequencies of naïve (T_N_: CD62L^+^CD44^−^), (**G**) central memory (T_CM_: CD62L^+^CD44^+^), (**H**) and effector memory (T_EM_: CD62L^−^CD44^+^) subsets within CD3^+^CD8^+^ T cells. (**I**) Relative distribution of CD8^+^ T_N_, T_CM_, and T_EM_ subsets, expressed as proportions of their combined total after Tulahuen HP stimulation. Each symbol represents an individual mouse; open circles correspond to females and filled circles to males. Data are presented as individual values with mean ± SEM. Statistical analysis was performed using two-way ANOVA followed by Sidak’s multiple-comparisons test. Asterisks indicate statistically significant differences (* *p* < 0.05; ** *p* < 0.01; *** *p* < 0.001).

The frequencies of CD4^+^ and CD8^+^ naïve (T_N_: CD62L^+^CD44^−^), central memory (T_CM_: CD62L^+^CD44^+^), and effector memory (T_EM_: CD62L^−^CD44^+^) T cell populations were analyzed by flow cytometry in splenocytes obtained, using the gating strategy depicted in Figure 4A. Splenocytes were re-stimulated in vitro with the N-Tc52/TSKb20 chimeric protein, the TSKb20 peptide, or the Tulahuen HP. No sex-related differences were observed in the CD4^+^ memory T cell subsets. The frequency of the CD4^+^ naïve subpopulation remained largely stable across conditions. However, following stimulation with Tulahuen HP, vaccinated animals displayed a significant reduction in T_N_ subset compared with non-vaccinated controls (64% vs. 77% respectively; *p* < 0.01) (Figure 4B). Regarding the CD4^+^ memory compartment, vaccinated animals presented a significant higher proportion of T_CM_ cells following stimulation with the *T. cruzi* protein homogenate compared with non-vaccinated animals (2.5% vs. 1.06% respectively; *p* < 0.05) (Figure 4C). Likewise, stimulation with the TSKb20 peptide or Tulahuen HP resulted in increased frequencies of CD4^+^ T_EM_ cells in vaccinated mice relative to non-vaccinated controls (4.3% vs. 2.4%, *p* < 0.01, and 4.7% vs. 2.6%, *p* < 0.05, respectively) (Figure 4D). Overall, the relative distribution of the average percentages of CD4^+^ T cell subpopulations was compared between non-vaccinated and vaccinated groups following re-stimulation with Tulahuen HP. As shown in Figure 4E, vaccinated animals exhibited a lower proportion of CD4^+^ T_N_ cells compared to non-vaccinated mice (90.4% vs. 94.4% respectively), a greater proportion of CD4^+^ T_CM_ cells (3.4% vs. 1.4%) and a higher frequency of CD4^+^ T_EM_ cells (6.1% vs. 3.2%) (Figure 4E).

As observed for CD4^+^ memory T cell subsets, no evidence of sex-based variation was found in CD8^+^ memory T cell subsets. The frequency of CD8^+^ naïve cells was significantly lower in vaccinated animals than in non-vaccinated mice following stimulation with TSKb20 (36% vs. 75%; *p* < 0.001) or the *T. cruzi* homogenate (35% vs. 78%; *p* < 0.001) (Figure 4F). Although no significant differences were observed in CD8^+^ T_CM_ cells across groups or stimulation conditions, vaccinated animals consistently showed a trend toward increased frequencies in response to all antigenic stimuli (Figure 4G). Furthermore, vaccinated mice exhibited a significantly higher proportion of T_EM_ cells than non-vaccinated controls following re-stimulation with either N-Tc52/TSKb20 or TSKb20 (10% vs. 7% and 9% vs. 5%, respectively; *p* < 0.05). Notably, Tulahuen HP stimulation induced a significant increase in T_EM_ cells in splenocytes from vaccinated mice compared with non-vaccinated mice (11% vs. 5%; *p* < 0.001) (Figure 4H). Analysis of the relative distribution of CD8^+^ T cell memory compartments after re-encounter with Tulahuen HP revealed that vaccinated animals exhibited higher proportions of T_CM_ (33.5% vs. 17.3%) and T_EM_ (15.9% vs. 5.4%) cells, accompanied by a reduction in the naïve subset (50.6% vs. 77.3%), compared with non-vaccinated mice (Figure 4I).

## 4. Discussion

Defining vaccine efficacy remains a major challenge, largely depending on the identification of reliable immunological correlates of protection. As described by Plotkin and colleagues, these correlates are measurable immune parameters that statistically associate with protection following vaccination [45]. While antibody titers have served as practical surrogates of protection for many licensed vaccines, such correlates remain poorly defined for several major infectious diseases, including malaria, tuberculosis, and CD [46,47]. In these infections, pathogens undergo intracellular stages during their life cycle, rendering T cell-mediated responses essential for their elimination [48]. As a result, reliance on antigen-specific antibody responses alone provides an incomplete measure of vaccine efficacy. In the case of *T. cruzi* infection, a comprehensive understanding of the interplay between humoral and cellular immune responses is critical for defining the mechanisms that contribute to protection and for guiding the identification of relevant correlates of protective immunity. In this context, the generation of long-term immunological memory emerge as a central determinant of successful vaccination [40]. Building on this framework, we conducted a comprehensive long-term evaluation of the quality, durability, and phenotype of the memory immune responses elicited by the N-Tc52/TSKb20 chimera in a murine model. By simultaneously interrogating both humoral and cellular immune compartments at extended time points following vaccination, this study provides critical insights into the persistence, functionality, and overall competence of vaccine-induced immunity.

At extended time points post-immunization, antigen-specific antibodies remained readily detectable, indicating the persistence of humoral immune memory. This sustained serological response is consistent with the establishment of long-lived plasma cell-mediated memory within the bone marrow [49]. Generated during germinal center reactions, these cells continuously secrete high-affinity antibodies in the absence of antigen and constitute the principal source of circulating serum antibodies [50,51,52]. In humans, plasma cells can persist for weeks, months, or potentially even longer [53]. Although long-lived plasma cells, memory B cells, affinity maturation, and endpoint antibody titers were not directly evaluated in this study, the continued detection of antigen-specific antibodies nearly six months after immunization supports the persistence of long-term humoral immunity.

The persistent predominance of IgG2c over IgG1 reflects a durable Th1-skewed humoral response. Consistent with previous reports, IgG2c has been associated with improved control of intracellular pathogens, including *T. cruzi*, contributing to more effective parasite clearance [54,55,56,57,58]. These effects are attributed to the enhanced effector functions of IgG2c antibodies, such as Fc receptor engagement, opsonization, and complement-mediated lysis [59,60,61]. In agreement with this, sera from vaccinated mice retained trypanolytic activity, demonstrating the persistence of functionally active antibodies capable of targeting extracellular parasites. Beyond their direct role in limiting parasite dissemination, antibodies can also modulate cellular immunity [62]. In intracellular infections, including those caused by *Chlamydia* and *Mycobacterium* species, IgG2a/c antibodies promote antigen uptake and presentation by antigen presenting cells, thereby enhancing T cell responses and reinforcing Th1-type immunity [62]. Consistent with this, the absence of mature B cells in *T. cruzi* infection has been associated with impaired CD4^+^ Th1 and CD8^+^ memory T cell responses, supporting a critical role for B cells in shaping T cell immunity [63,64]. Therefore, the persistence of a Th1-bias humoral response may extend beyond sustained antibody functionality and contribute to the long-term modulation of cellular immune responses. Such crosstalk between humoral and cellular immunity is especially relevant in *T. cruzi* infection, where durable CD4^+^ and CD8^+^ T cell responses have been consistently associated with immune protection and parasite control. To assess the persistence of T cell effector functions, antigen recall assays were conducted 170 days after the final immunization. T cell activation was evaluated through CD25 and CD69 co-expression, while IFN-γ and TNF-α secretion served as indicators of Th1-associated functionality. The sustained responsiveness of TSKb20-specific CD8^+^ T cells was evidenced by the coordinated expression of these activation markers together with cytokine production following antigen re-encounter. Collectively, these parameters are consistent with the maintenance of a functionally responsive T-cell population long after vaccination [44,65]. IFN-γ and TNF-α are pro-inflammatory cytokines that have been consistently associated with immune control of *T. cruzi* infection. In particular, CD8^+^ T cells specific for TSKb20 or other immunodominant epitopes are recognized as key mediators of protective responses against the parasite [42,66,67,68,69,70]. Supporting this notion, vaccination with a combination of DNA vaccine encoding trans-sialidase and IL-15 enhanced the proliferative and effector functions of trans-sialidase-specific CD8^+^ T cells, with these features persisting for six months post-vaccination. Importantly, the study demonstrated that these cells alone were sufficient to confer protection upon parasite challenge [71]. Together, these findings suggest that the maintenance of TSKb20-specific effector functions months after vaccination reflects the establishment of a durable and functionally competent T cell memory response.

We next evaluated memory T cell phenotypes based on the expression of CD44 and CD62L. Both CD4^+^ and CD8^+^ T cells exhibited a trend toward increased frequencies of T_CM_ and T_EM_ subsets. This increase was particularly pronounced in CD8^+^ T_EM_ cells following TSKb20 stimulation and, even more markedly, with the *T. cruzi* protein homogenate. In addition, vaccinated animals exhibited approximately two-fold higher frequencies of CD8^+^ T_CM_ cells than non-vaccinated controls. The establishment of a durable memory T-cell compartment has been identified as an important feature of vaccine-induced immunity against *T. cruzi* and has been reported in other protective vaccine formulations [72,73,74]. T_CM_ cells are characterized by high proliferative capacity upon antigen re-exposure and greater longevity than T_EM_ cells, enabling the long-term maintenance of immune memory and sustained protective immunity [75]. In contrast, T_EM_ cells display a more terminal phenotype and are capable of rapidly producing cytokines such as IFN-γ and IL-2 and harboring preformed perforin, thereby providing a faster but potentially less durable response [76]. However, the advantage of T_CM_ compared with T_EM_ responses remains unclear. Although T_CM_ cells have been linked to superior long-term protection against systemic infections [77,78], this phenotype alone does not necessarily indicate a higher-quality immune response [79]. In contrast, CD8^+^ T_EM_ cells can respond rapidly upon antigen recall, a feature that is considered crucial for early protection against *T. cruzi* [80]. Consistent with these observations, growing evidence suggests that the protective efficacy of heterologous prime–boost vaccination strategies is closely linked to their ability to generate strong memory CD8^+^ T cell responses [72,81,82]. In contrast, the induction of comparable cellular memory has generally been more difficult to achieve using recombinant protein-based vaccine platforms [83]. Notably, a recent comparative study reported that both heterologous prime–boost and adjuvanted recombinant protein-based vaccination strategies elicited CD8^+^ TEM cell responses in female mice that persisted for at least 90 days after the final immunization and were associated with protection against *T. cruzi* challenge [26]. In this sense, CD8^+^ T_EM_ cells have emerged as a critical population linked to long-term protective immunity against parasitic infections [84] and may represent a relevant correlate of protection for evaluating vaccine efficacy against *T. cruzi*. Several studies have associated the long-term persistence of antigen-specific CD8^+^ T cell responses with protection against *T. cruzi* infection. For example, Gupta and Garg reported the maintenance of CD4^+^ and CD8^+^ T_CM_ and T_EM_ populations up to 120 days after immunization, which correlated with robust immune responses and protection upon challenge [85]. Likewise, heterologous prime–boost vaccination with AdASP-2 induced long-lasting effector CD8^+^ T cells capable of producing IFN-γ and TNF-α up to 98 days after immunization [72]. A more recent study using the chimeric protein TRASP demonstrated the persistence of IFN-γ^+^ CD8^+^, CD8^+^ T_EM_, and CD8^+^ T_CM_ populations at 90 days post-immunization, with both responses associated with protection against *T. cruzi* challenge [26]. Collectively, these findings demonstrate that, even 170 days after immunization, the N-Tc52/TSKb20 antigen sustains a durable capacity to elicit antigen-specific cellular responses. Notably, this response is not restricted to vaccine-derived antigens but extends to parasite-derived proteins, underscoring the long-term breadth and relevance of the induced immune memory. Nevertheless, as stimulation with *T. cruzi* protein homogenate constitutes only an in vitro approximation of infection, the biological relevance of these findings must be validated in vivo. To this end, challenge experiments with virulent parasites at late post-immunization time points will be essential to determine whether the observed immune responses translate into effective protection. Furthermore, future studies are needed to more thoroughly investigate memory T cell compartments, including assessments of exhaustion markers, proliferative capacity, cytotoxicity, and poly-functionality, to directly evaluate the effector capabilities of these cell types essential for effective vaccination.

Given the growing recognition of sex as a biological variable influencing immune responses, the inclusion of both females and males in preclinical vaccination models is essential to accurately evaluate vaccine efficacy [86,87]. Notably, sex-dependent differences were detected in some immunological parameters evaluated in this study. In contrast, female mice exhibited significant IL-10 production in response to TSKb20 and *T. cruzi* protein stimulation, whereas male mice maintained IL-10 responses at levels similar to those observed in non-vaccinated animals. As a pivotal regulatory cytokine, IL-10 is critical for shaping immune homeostasis [88], and its sustained production suggests that, even at late time points post-immunization, vaccination in females may promote a more balanced and tightly regulated immune response. This observation is particularly relevant considering that females are generally more prone to developing autoimmune diseases [89]; thus, the induction of a balanced immune response after vaccination represents a desirable feature to minimize the risk of rare adverse events. Additionally, subtle sex-dependent differences were observed in the distribution of T cell subsets, with females displaying higher CD4^+^ and lower CD8^+^ T cell frequencies and males exhibiting the inverse pattern. Despite suggesting distinct immunological tendencies, these differences did not result in detectable changes in activation or functional responses upon antigenic stimulation, underscoring their limited influence on the overall quality of the immune response. In murine models, males are consistently more susceptible to infection, displaying higher parasitemia, decreased survival, and more severe clinical outcomes [90,91], a trend that has also been observed in some human studies [92,93]. In this context, a vaccination-induced profile characterized by reduced IL-10 production together with an increased proportion of CD8^+^ T cells could theoretically contribute to improved parasite control in males. Nevertheless, these observations should be interpreted cautiously given the relatively small number of animals included in the sex-specific analyses. Further studies are required to experimentally validate this hypothesis and to determine whether these sex-associated immunological differences ultimately influence vaccine efficacy or safety.

Reflecting this complexity, evidence on sex-based differences in vaccine-induced immunity remains variable and context-dependent [87]. While females are often reported to develop more immunogenic Th1-skewed responses following vaccination [94,95], this trend is not consistent across studies. In fact, males have been shown in some cases to mount more pronounced Th1 responses, with higher IFN-γ and IL-2 production and lower levels of IL-10 and IL-4 [96]. These inconsistencies are particularly evident in vaccines against intracellular pathogens, where cellular immunity is a key determinant of protection. For instance, BCG vaccination against *Mycobacterium tuberculosis* has been shown to induce CD8^+^ T_CM_ and CD4^+^ T_EM_ responses in females, correlating with improved clinical outcomes [97]. In contrast, other studies have reported sex-dependent differences in vaccine-induced immunity, showing that modified BCG formulations preferentially promote Th1-type responses in males, while BCG Pasteur enhances TNF-α-producing CD4^+^ and CD8^+^ T-cell populations in females up to 60 days post-vaccination [98]. Similar sex-dependent effects have been described in malaria, with RAS vaccines eliciting stronger antibody-mediated protection in females, whereas RAS-based prime-and-trap strategies induce lower frequencies of protective hepatic memory CD8^+^ T cells in males [99,100]. Despite these advances, data on sex-specific immune responses in the context of *T. cruzi* vaccination remain scarce, with only one recent study evaluating sex-based differences using an orally administered recombinant TS protein in BALB/c mice. In that study, females displayed stronger antibody and cytokine responses while males showed enhanced T cell-mediated immunity. Despite these sex-dependent immunological differences, both groups achieved comparable protection following oral challenge [91]. These findings, together with our observations, highlight the critical need to incorporate sex as a biological variable in preclinical vaccine research to better inform the design of effective and safe immunization strategies.

## 5. Conclusions

Taken together, these findings demonstrate that immunization with the N-Tc52/TSKb20 chimera promotes the establishment of a durable and functional antigen-specific humoral and cellular immune response in both female and male mice. Specifically, this vaccine induced a persistent Th1-biased humoral response with trypanolytic activity, together with durable TSKb20-specific CD8^+^ T cell responses characterized by IFN-γ and TNF-α production. These responses were accompanied by the maintenance of a robust CD8^+^ T_EM_ population capable of recognizing parasite-derived antigens long after immunization. Collectively, these findings support the persistence of a functionally competent memory immune compartment. Nevertheless, the lack of a long-term in vivo challenge experiment represents an important limitation of this study. While the results demonstrate the durability of vaccine-induced immune responses, additional studies will be required to establish whether these responses confer protection against *T. cruzi* infection at extended time points and across both sexes. Nevertheless, in the absence of well-established correlates of protection for *T. cruzi* vaccine efficacy, our findings support the use of durable functional immune responses as an important parameter for evaluating vaccine candidates. In addition, vaccinated female mice showed a trend toward higher IL-10 production following antigen re-stimulation than males. Although these observations were based on a limited number of animals, they suggest a potential influence of sex on vaccine-induced immunity and reinforce the importance of considering sex as a biological variable in future studies. Overall, given the scarcity of long-term immunological studies in CD vaccinology, this work provides valuable insights into the durability of vaccine-induced immune responses and further supports the potential of N-Tc52/TSKb20 as a promising vaccine candidate. Future studies will be required to determine whether these long-lasting immune responses translate into protection against *T. cruzi* at extended time points and contribute to the attenuation of clinical disease manifestations.

## Data Availability

The data that support the findings of this study are available from the corresponding author, upon reasonable request.
